# Residency training programs in anesthesiology, intensive care and emergency medicine: from curriculum to practice

**DOI:** 10.3389/fmed.2024.1386681

**Published:** 2024-06-26

**Authors:** Claudiu Barsac, Alina Petrica, Diana Lungeanu, Adina Maria Marza, Ovidiu Bedreag, Marius Papurica, Cosmin Iosif Trebuian, Mihai O. Botea, Ovidiu Alexandru Mederle, Dorel Sandesc

**Affiliations:** ^1^Department of Surgery, “Victor Babes” University of Medicine and Pharmacy, Timisoara, Romania; ^2^Clinic of Anesthesia and Intensive Care, “Pius Brinzeu” Emergency Clinical County Hospital, Timisoara, Romania; ^3^Emergency Department, “Pius Brinzeu” Emergency Clinical County Hospital, Timisoara, Romania; ^4^Center for Modeling Biological Systems and Data Analysis, “Victor Babes” University of Medicine and Pharmacy, Timisoara, Romania; ^5^Department of Functional Sciences, “Victor Babes” University of Medicine and Pharmacy, Timisoara, Romania; ^6^Emergency Department, Emergency Clinical Municipal Hospital, Timisoara, Romania; ^7^Department of Surgery, Faculty of Medicine and Pharmacy, University of Oradea, Oradea, Romania; ^8^Oradea County Clinical Emergency Hospital, Oradea, Romania; ^9^Pelican Clinic, Medicover Hospital, Oradea, Romania

**Keywords:** anesthesia, intensive care, critical care, residency program, practical skills, mentorship, simulation in medical training, medical education

## Abstract

Residency programs in anesthesiology and intensive care (AIC), and emergency medicine (EM) continually evolve to ensure well-prepared trainees for these critical fields of healthcare. The objective of this study was to collect comprehensive feed-back from AIC and EM residents, comprising opinions and attitudes on: curriculum and structure of the residency program; scope of training environment, opportunities and complexity; training guidance and mentorship; teaching approach. An anonymous online cross-sectional survey was conducted among AIC and EM trainees during December 2023–January 2024 and June 2023–July 2023, respectively. Two hundred and thirty-five answers were collected: 137 (73/64 female/male) and 98 (55/43 female/male) respondents from the AIC and EM programs, respectively. Overall feed-back was equivalent for both residency programs, with differences related to the distinct characteristics of each medical specialty. The main issues identified across the programs were the need to improve and diversify the teaching approaches, with trainees' strong desire for more professional guidance, mentoring, and constant feed-back. The findings would inform decision-making beyond current residency programs in these critical care specialties, highlighting the need to design solutions for interactive and highly immersive educational experiences, such as simulation, augmented reality or virtual reality.

## 1 Introduction

Workforce-related challenges are widespread in the healthcare industry, including the fields of anesthesiology and intensive care (AIC), and emergency medicine (EM). Previous efforts aimed at enhancing retention in these specialties have addressed the staffing problem, but they resulted in mere changes rather than outright improvement (1). After a global pandemic that put major strains on AIC and EM worldwide, the two specialties are currently facing a lack of resident doctors in our country as a result of a low interest in choosing such a demanding specialty at the residency entry examination or later dropping out of the residency programs (2).

Amidst the pandemic, emergency departments (EDs) across the world served as the first line of defense being shouldered by the intensive care units (ICUs). This resulted in physicians working tirelessly, often with limited resources over extended hours and without adequate support for young doctors, with a toll on their empathy and commitment in assuming the required responsibilities (3, 4). This stress, combined with curricula or training that do not meet expectations, might be behind the declining interest in the two fields, or this trend could simply be a diversion of interest and attention from critical patients.

Research has been published on how anesthesia training is conducted in different countries (5), but there is scarcity of comparative studies to examine the structure and requirements of graduate medical education for various critical care specialties. While each field has its unique focus and expertise, there is often significant overlap in the care provided by anesthesiologists, intensivists, and emergency physicians, particularly in critical or emergency situations. Their collaboration ensures seamless patient care from the ED to the operating room and the ICU, thus enhancing patient outcomes and improving the overall healthcare delivery. These specialties involve the management and care of critically ill or injured patients. Anesthesiologists, intensivists, and emergency physicians are trained to handle high-stress situations and make rapid decisions to stabilize patients and provide appropriate treatment. They often require close collaboration with a large variety of healthcare professionals, including surgeons, nurses, respiratory therapists, and other specialists. Effective teamwork is crucial to ensure comprehensive care.

The European curriculum for anesthesiology residency places a strong emphasis on acquiring the knowledge and skills necessary for providing care to patients throughout the perioperative period. This includes conducting pre-operative assessments, managing patients during surgery, and effectively addressing post-operative pain, therefore residents undergo extensive training in anesthesia techniques, pharmacology, critical care, and pain management (6).

The Romanian curriculum for AIC residency comprises a 5-year rotation scheme in different settings, namely in ICUs across different hospitals to cover a wide range of pathologies, such as cardiovascular, respiratory, renal, digestive, neurological, trauma, burns, pediatrics, and geriatrics. The anesthesia rotations cover specific perioperative challenges from all types of surgeries: general; vascular; urology; gynecology and obstetrics; cardio-thoracic; orthopedics; ear, nose, throat; and neurosurgery. Alongside these rotations, continuous theoretical support is given to AIC residents in terms of courses and workshops on different topics, such as basic and advanced resuscitation skills, peripheral and central line insertion techniques, mechanical ventilation, difficult airway management, neuraxial techniques or peripheral nerve blocks (7).

On the other hand, EM residency programs are specifically designed to prepare physicians for the fast-paced and unpredictable ED environment. EM residents are trained to handle a wide range of acute medical conditions and trauma cases. Their curriculum focuses on developing skills in resuscitation, stabilization, diagnostic evaluation, and acute management of emergencies (8). EM is officially acknowledged as a distinct medical specialty in 16 European countries, necessitating a minimum of 5-year training. Additionally, 33 European countries have adhered to the “European Doctors' Directive” (9), either adopting a 5-year training regimen or opting for alternative programs (for example, a 4-year curriculum, and two to 3-year specialized training for EM as a supra-specialty). Furthermore, one of the main important points emphasized at the beginning of the recommendations highlights the differentiation between EM field and emergency medical care provided by practitioners in various other specialties. A different approach is seen in Australia and New Zealand, where the EM residency program takes seven years, comprising 2-year basic training, 1-year provisional training, and 4-year advanced training (10).

The Romanian curriculum for EM residency spans 5 years designed to provide a well-rounded experience. Each year of residency is structured into modules, with modules specifically focused on EM (e.g., emergency admission unit and mobile resuscitation unit) being the longest and most comprehensive. The modules covering adult and pediatric AIC take almost 17 months and are a crucial part of the emergency physician's training; therefore, right in the 1^st^ year of EM residency, a dedicated 5-month module is designed to enhance practical skills in resuscitation and critical patient management. Additionally, other essential modules encompass various surgical specialties (e.g., general surgery, orthopedics, thoracic surgery, plastic surgery, obstetrics, and gynecology), cardiology, pediatrics, and neonatology (11).

There is a certain degree of overlapping between AIC and EM, but it is paramount for the residency curricula to tailor the training in order to meet the distinct demands and challenges of each specialty. An analysis encompassing the residency curricula of AIC and EM can provide valuable insights and benefits. A deeper understanding of the similarities and differences between such training programs can help educators, administrators, and policymakers make informed decisions about curriculum design and resource allocation. Sharing experiences can lead to collaborative efforts in developing joint educational initiatives, interdisciplinary training, or shared resources, ultimately benefiting the healthcare services beyond these two specialties.

In the context of existing residency curricula for these two critical care specialties, the primary objective of this study was to collect comprehensive feed-back from trainees. The feed-back would comprise opinions and attitudes on: curriculum and structure of the residency program; the training environment scope, opportunities and complexity; training guidance and mentorship; teaching approach. The secondary objectives were: (a) to identify common patterns in trainees' opinions and attitudes and examine their relevance; (b) to establish the relevance of the differences and their subsequent usability/applicability in adjusting the residency programs; (c) to estimate the response rate and have a basis for designing further multicenter longitudinal analysis of post-graduate training in AIC and EM.

## 2 Materials and methods

### 2.1 Study design and participants

An online cross-sectional survey was employed for data collection: an anonymous web-based questionnaire (implemented using Google Forms) was distributed through professional channels and networks (e.g., WhatsApp groups and professional e-mail groups) to the residents of AIC and EM during December 2023–January 2024 and June 2023–July 2023, respectively. The main 11 university centers in Romania were targeted: Arad, Brasov, Bucuresti, Cluj, Constanta, Craiova, Iasi, Oradea, Sibiu, Targu-Mures, and Timisoara. Each questionnaire was closed after three consecutive days with no answer. The questionnaire, provided in full as Supplementary material of this manuscript (Supplementary file 1), is adapted after EUSEM recommendations (12); it started with information about the study's objectives and measures taken to assure the personal data protection. For each individual, actual data collection proceeded after informed consent had been granted (a required confirmation was included as the first item).

The survey covered the four areas targeted by the main objective: (a) curriculum and structure of the residency program; (b) training environment, scope, opportunities and complexity; (c) training guidance and mentorship; (d) teaching and assessment approach. They were implemented as 11 distinct sections in the questionnaire: (1) induction to the residency program, (2) overall curriculum design, (3) work environment, (4) mentorship, (5) teaching and educational activities, (6) evaluation of residents, (7) research, (8) interdepartmental support, (9) resources and infrastructure, (10) program director, and (11) overall opinion, suggestions and recommendations.

**Figure 1:**
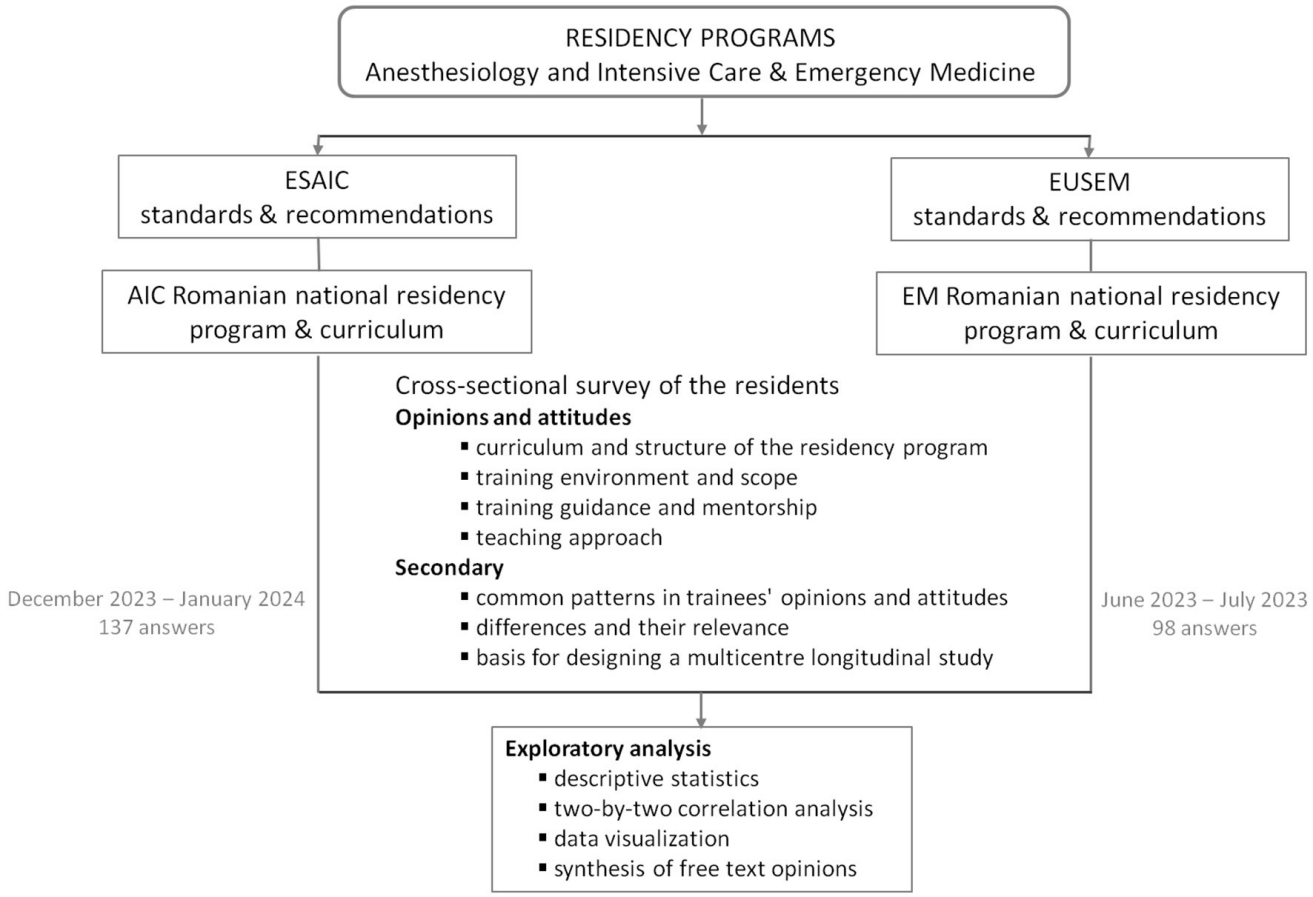
Flow chart of study. AIC, Anesthesiology and Intensive Care; EM, Emergency Medicine; ESAIC, European Society of Anaesthesiology and Intensive Care; EUSEM, European Society for Emergency Medicine.

Figure 1 shows the study flow diagram.

The questionnaire collected categorical and numerical data; 42 questions on five-point Likert-type scales probed trainees' perception of the way the residency curriculum is conveyed into practice. This paper is focused on the quantitative data; however, the last section of the questionnaire also included open-ended questions trying to capture the residents' overall opinion. The face validity of questionnaire's items (namely, their relevance to this project) was assured by a Delphi technique in the development process; no prior formal validation was conducted. All questions were compulsory.

The study was conducted in accordance with the Declaration of Helsinki, and the protocol was approved by the Ethics Committee of “Pius Branzeu” Emergency County Hospital no 432/16.01.2024.

### 2.2 Data analysis

Reliability of the scales' measurements was assessed based on Cronbach's alpha (the value was calculated for each sub-scale corresponding to the issues addressed by the main objective). Values of Cronbach's alpha > 0.8 were considered to indicate good internal consistency. Intraclass correlation coefficient (ICC) with 95% confidence interval (CI) was also calculated as an index of the extent to which measurements can be replicated. Values higher than 0.5 for ICC were considered as a further proof of moderate to good reliability.

For each subscale, analysis was separately conducted on each item and a subsequent composite measure was calculated as the arithmetic mean of the respective subscale indicators. This aggregated metric helped further quantitative analysis and comprehensive comparison of the two residency programs. Radar plots with the four aggregated metrics were used for data visualization.

Exploratory statistical analysis was conducted. Descriptive statistics included: the observed frequency counts and percentages for categorical variables; median with inter-quartile range (IQR) with Tukey's hinges and mean with standard deviation (SD) for numerical variables, irrespective of their distribution.

Univariate non-parametric Mann-Whitney U statistical test was applied to compare the distribution of numerical data across two groups (i.e., corresponding to the residency programs). The chi-square statistical test (either asymptotic, Fisher's exact test, or Monte-Carlo simulation with 10,000 samples) was applied to check the statistical significance of the association between the categorical variables. To explore covariance between various subscale scores, two-by-two non-parametric Spearman correlation analysis was conducted and scatter plots were used for data visualization.

The statistical analysis was conducted at 5% level of statistical significance (95% level of confidence). All reported probability values are two-tailed.

Data analysis was performed with the statistical software IBM SPSS v. 20 and R v. 4.3.1 packages (including “fmsb” v. 0.7.5 and “ggplots” v. 3.1.3).

## 3 Results

### 3.1 General characteristics of respondents

The survey gathered information from 235 trainees: 137 AIC residents and 98 EM residents. One hundred and twenty-eight were female medical doctors (73 AIC and 55 EM) and 107 were males (64 AIC and 43 EM). Answers were received from each of the 11 residency centers, with inhomogeneous geographical distribution across the two specialties. Based on residency places during the period 2019–2022 (744 AIC and 668 EM) as a reference and a 75% retention rate in residency programs (i.e., 25% drop-out in these specialties), the raw rates of feed-back return were 24.55% and 19.56% for AIC and EM, respectively. An overall response rate of approximately 20% in both specialties was inferred.

### 3.2 Feed-back from trainees

Table 1 shows the trainees' opinion regarding the curriculum and structure of the program they follow and their perceived efficiency of the initial orientation into the program. Although the difference in overall grade on orientation and curriculum of the residency program is statistically significant, the actual difference is less than half a point on the five-point Likert scale. However, there is a more substantial 0.6-point mean difference for the scale item regarding the clarity of the curriculum objectives in favor of the EM program. Even more, regarding the median (IQR), this difference is a full point on the five-point Likert scale. This discrepancy is apparent in the qualitative variable concerning the content of the curriculum: 42.4% of the AIC residents believe there is no specific curriculum or they do not have knowledge of such a formal structure of the residency program, in contrast with only 12.2% of the EM residents having the same opinion.

**Table 1:** Opinions on curriculum and structure of the residency program.

**Variable**	**AIC (*N =* 137)**	**EM (*N =* 98)**	***p*-value^a, b^**
**Qualitative outcomes**			
Curriculum content^a^			< 0.001^**^
EUSEM or ESAIC curriculum	44 (32.1%)	37 (37.8%)	
National curriculum	35 (25.5%)	49 (50.0%)	
There is no specific curriculum	36 (26.3%)	11 (11.2%)	
I don't know	22 (16.1%)	1 (1.0%)	
**Quantitative outcomes**			
Scale Cronbach's alpha: 0.899 (*N =* 5 items)			
Scale intraclass correlation coefficient: 0.640, 95%CI (0.587; 0.691), *p* < 0.001^**^			
Initial guidance for the entire training program^b^	3.11 ± 1.247 3 (2–4)	3.44 ± 1.21 3 (3–4)	0.039^*^
Objectives of the entire training program were clearly stated at the beginning^b^	2.84 ± 1.29 3 (2–4)	3.29 ± 1.362 3 (2–5)	0.012^*^
Orientation on the departments, facilities and teaching was offered at the beginning^b^	3.12 ± 1.374 3 (2–4)	3.31 ± 1.311 3 (2–5)	0.334
Objectives of the curriculum are clear^b^	3.15 ± 1.254 3 (2–4)	3.79 ± 1.028 4 (3–5)	< 0.001^**^
Structure of the curriculum is clear^b^	3 ± 1.26 3 (2–4)	3.52 ± 1.048 3 (3–4)	0.002^**^
Overall grade for orientation and curriculum of the residency program^b^	3.0453 ± 1.0973 3 (2.2–3.8)	3.4673 ± 0.9831 3.4 (2.8–4.4)	0.006^**^

^a^Categorical variable; count (percentage); asymptotic chi-square test or Monte-Carlo simulation. ^b^Numerical variable (from 1-very little so, to 5-strongly agree); mean ± standard deviation; median (IQR), non-parametric Mann-Whitney test. AIC, anesthesiology and intensive care; CI, confidence interval; EM, emergency medicine; ESAIC, European Society of Anaesthesiology and Intensive Care; EUSEM, European Society for Emergency Medicine; IQR, inter-quartile range. Level of statistical significance: ^*^p < 0.05; ^**^p < 0.01.

Table 2 presents the trainees' opinion on the training environment: its scope, infrastructure, adequacy, learning opportunities, and staff preparedness or training for providing the necessary support.

**Table 2:** Opinions on training environment, scope, opportunities and complexity.

**Variable**	**AIC (*N =* 137)**	**EM (*N =* 98)**	***p*-value^a, b^**
**Qualitative outcomes**			
Number of training environments^a^			< 0.001^**^
1–2	8 (5.8%)	74 (75.5%)	
3–4	19 (13.9%)	17 (17.3%)	
5 or more	110 (80.3%)	7 (7.1%)	
**Quantitative outcomes**			
Scale Cronbach's alpha: 0.899 (*N =* 10 items)			
Scale intraclass correlation coefficient: 0.472, 95%CI (0.421; 0.526), *p < * 0.001^**^			
Training sites are adequate in terms of educational content^b^	3.39 ± 1.197 3 (3–4)	3.66 ± 1.121 4 (3–5)	0.122
Adequate complexity of cases encountered in the department^b^	4.37 ± 0.840 5 (4–5)	4.19 ± 0.960 4 (4–5)	0.18
Adequate opportunity for practical skills development^b^	3.47 ± 1.237 4 (3–4)	3.72 ± 1.023 4 (3–5)	0.163
Adequate pace for progressive responsability development^b^	3.54 ± 1.182 4 (3–5)	3.92 ± 1.071 4 (3–5)	0.014^*^
Interdepartmental relationships^b^	3.01 ± 1.173 3 (2–4)	3.18 ± 1.059 3 (2–4)	0.251
Cross-department training relationships^b^	2.77 ± 1.279 3 (2–4)	2.84 ± 1.266 3 (2–4)	0.698
Cross-department curriculum compliance^b^	2.66 ± 1.251 3 (2–4)	2.54 ± 1.270 2 (2–3)	0.389
Staff support during cross-department training^b^	2.78 ± 1.229 3 (2–4)	3.43 ± 1.316 4 (3–4)	< 0.001^**^
Department-level adequate infrastructure for educational activities^b^	3.34 ± 1.244 3 (2–4)	3.53 ± 1.186 3.5 (3–5)	0.272
Hospital-level adequate infrastructure for educational activities^b^	3.16 ± 1.307 3 (2–4)	3.22 ± 1.231 3 (2–4)	0.748
Overall grade for environment adequacy^b^	3.248± 0.8728 3.2 (2.5–3.9)	3.425 ± 0.8367 3.4 (2.8–4.1)	0.211

^a^Categorical variable; count (percentage); asymptotic chi-square test or Monte-Carlo simulation. ^b^Numerical variable (from 1-very little so, to 5-strongly agree); mean ± standard deviation; median (IQR), non-parametric Mann-Whitney test. AIC, anesthesiology and intensive care; CI, confidence interval; EM, emergency medicine; IQR, inter-quartile range. Level of statistical significance: ^*^p < 0.05; ^**^p < 0.01.

There is no significant difference in the overall grade for environment adequacy, but the staff is perceived as being less supportive in the AIC specialty (0.65-point mean difference between the programs, with a full point difference between the median values). Additionally, the development of progressive responsibility is perceived by AIC trainees as being significantly less adequate, but the mean difference is < 0.4-point compared to the EM residents. This small discrepancy should be regarded in the context of the actual design and organization of the training facilities of the two residency programs: five or more distinct wards or environments for AIC, compared to two up to four in EM.

Table 3 show the opinions on training guidance, mentoring, and perceived role of the residency program's director. There is little difference between the two programs (0.33-point mean difference on the Likert scale) in the overall grade for guidance and mentorship (Table 3). The difference seems to be related to the adequacy of the available mentors' number and availability (approximately 0.5-point mean difference on the Likert scale; Table 3). The qualitative outcomes in Table 3 reveal a better perception regarding the mentoring and the role of the program director among the EM trainees, which might also be related to the number of distinct wards or training environments (i.e., a consequence of higher number of residency rotations in the AIC program).

**Table 3:** Opinions on training guidance and mentorship: qualitative and quantitative outcomes.

**Variable**	**AIC (*N =* 137)**	**EM (*N =* 98)**	***p-*value^a^**
**Qualitative outcomes**			
Residency mentors^a^			0.021^*^
No	33 (24.1%)	11 (11.2%)	
Yes	88 (64.2%)	80 (81.6%)	
I don't know	8 (5.8%)	2 (2.0%)	
NA	8 (5.8%)	5 (5.1%)	
Mentors are trained for teaching^a^			0.01^*^
No	10 (7.3%)	2 (2.0%)	
Yes	51 (37.2%)	56 (57.1%)	
I don't know	41 (29.9%)	25 (25.5%)	
NA	35 (25.5%)	15 (15.3%)	
Mentors follow/assess the trainees' progress^a^			< 0.001^**^
Never	32 (23.4%)	17 (17.3%)	
Monthly	19 (13.9%)	39 (39.8%)	
1–2 times per year	54 (39.4%)	9 (9.2%)	
NA	19 (13.9%)	15 (15.3%)	
Other	13 (9.5%)	18 (18.4%)	
Teaching sessions with mentors^a^			< 0.001^**^
Never	14 (10.2%)	12 (12.2%)	
Monthly	36 (26.3%)	40 (40.8%)	
1–2 times per year	52 (38.0%)	11 (11.2%)	
NA	16 (11.7%)	35 (35.7%)	
Other	19 (13.9%)	–	
**Quantitative outcomes**			
Scale Cronbach's alpha: 0.929 (*N =* 8 items)			
Scale intraclass correlation coefficient: 0.620, 95%CI (0.572; 0.669), *p < * 0.001^**^			
Mentors' number and availability are appropriate to the trainees' needs^b^	2.95 ± 1.374 3 (2–4)	3.44 ± 1.324 3 (2–5)	0.008^**^
Mentors and professional staff appropriately supervise trainees^b^	3.72 ± 1.2 4 (3–5)	4.07 ± 1.105 4 (3–5)	0.015^*^
Active bedside training is part of common activity^b^	2.91 ± 1.288 3 (2–4)	3.28 ± 1.361 3 (2–4)	0.03^*^
Program director provides continuous feed-back to trainees^b^	2.74 ± 1.323 3 (2–4)	3.23 ± 1.368 3 (2–4)	0.007^**^
Program director is effective^b^	3.36 ± 1.350 4 (2–5)	3.63 ± 1.295 4 (3–5)	0.128
Program director is required in the coordination of education and training^b^	3.30± 1.4 3 (2–5)	3.58 ± 1.251 4 (3–5)	0.145
Program director is required in the management of mentoring and trainees' rotation^b^	3.31 ± 1.413 4 (2–5)	3.46 ± 1.286 4 (3–5)	0.525
Program director is open and accessible to trainees^b^	3.68 ± 1.328 4 (3–5)	3.92 ± 1.298 4 (3–5)	0.128
Overall grade for guidance and mentorship^b^	3.246 ± 1.088 3.250 (2.375–4)	3.577 ± 1.050 3.688 (2.875–4.375)	0.016^*^

^a^Categorical variable; count (percentage); asymptotic chi-square test or Monte-Carlo simulation. ^b^Numerical variable (from 1-very little so, to 5-strongly agree); mean ± standard deviation; median (IQR), non-parametric Mann-Whitney test. AIC, anesthesiology and intensive care; EM, emergency medicine; NA, not applicable; CI, confidence interval; IQR, inter-quartile range. Level of statistical significance: ^*^p < 0.05; ^**^p < 0.01.

Tables 4 and 5 reveal a mixed perception of the teaching approach and assessment methods, with their frequency and accreditation. Table 6 presents quantitative opinions and perceptions of the training approaches, including formal education activities and opportunities to engage in applied translational research.

**Table 4:** Opinions on teaching approach (educational methods and instruments): qualitative outcomes.

**Variable**	**AIC (*N =* 137)**	**EM (*N =* 98)**	***p*-value^a^**
**Formal education activities**			
Courses^a^	96 (70.1%)	73 (74.5%)	0.458
Workshops^a^	100 (73%)	47 (48%)	< 0.001^**^
Discussions^a^	70 (51.1%)	71 (72.4%)	0.001^**^
Case presentations^a^	81 (59.1%)	62 (63.3%)	0.521
Simulations^a^	56 (40.9%)	3 (3.1%)	< 0.001^**^
Journal clubs^a^	14 (10.2%)	–	0.001^**^
Leadership training^a^	3 (2.2%)	3 (3.1%)	0.696
Other^a^	6 (4.4%)	6 (6.1%)	0.563
**Formal education frequency** ^a^			0.018^*^
Never	3 (2.2%)	5 (5.1%)	
Once per year	16 (11.7%)	5 (5.1%)	
Two times per year	27 (19.7%)	8 (8.2%)	
Every 4 months	29 (21.2%)	22 (22.4%)	
Every month	35 (25.5%)	40 (40.8%)	
Every week	27 (19.7%)	18 (18.4%)	
**One formal education activity which should be organized more often** ^a^			< 0.001^**^
Workshops for practical abilities	29 (21.2%)	28 (28.6%)	
Case-based debriefings	51 (37.2%)	18 (18.4%)	
Case presentations by residents	10 (7.3%)	2 (2%)	
Simulations (low to high fidelity)	38 (27.7%)	39 (39.8%)	
Journal clubs	–	1 (1%)	
Leadership training	5 (3.6%)	10 (0.2%)	
Other	4 (2.9%)	–	
**Additional courses for accreditation are required** ^a^	64 (46.7%)	49 (50%)	0.619

^a^Categorical variable; count (percentage); asymptotic chi-square test, Fisher's exact test or Monte-Carlo simulation. AIC, anesthesiology and intensive care; EM, emergency medicine. Level of statistical significance: ^*^p < 0.05; ^**^p < 0.01.

**Table 5:** Opinions on assessing methods and instruments: qualitative outcomes.

**Variable**	**AIC (*N =* 137)**	**EM (*N =* 98)**	***p*-value^a^**
**Evaluation/assessment methods**			
MCQ^a^	109 (82%)	70 (71.4%)	0.058
Short answer questions^a^	32 (23.5%)	33 (33.7%)	0.087
Structured clinical examination (OSCE)^a^	21 (15.3%)	37 (37.8%)	< 0.001^**^
Interview^a^	10 (7.3%)	12 (12.2%)	0.199
Other, simulations^a^	6 (4.4%)	11 (11.2%)	0.046^*^
**Assessments are scheduled**			
At the end of each year^a^	51 (37.2%)	15 (15.3%)	< 0.001^**^
At the end of each rotation^a^	33 (24.3%)	5 (5.1%)	< 0.001^**^
Occasionally ^a^	61 (45.5%)	66 (67.3%)	0.001^**^
Never^a^	8 (5.8%)	7 (7.1%)	0.687
Other^a^	3 (2.2%)	3 (3.1%)	0.696

^a^Categorical variable; count (percentage); asymptotic chi-square test, Fisher's exact test or Monte-Carlo simulation. AIC, anesthesiology and intensive care; EM, emergency medicine; MCQ, multiple choice questionnaire; OSCE, objective structured clinical examination. Level of statistical significance: ^*^p < 0.05; ^**^p < 0.01.

**Table 6:** Opinions on teaching and assessment: quantitative outcomes.

**Variable**	**AIC (*N =* 137)**	**EM (*N =* 98)**	***p*-value^a^**
Scale Cronbach's alpha: 0.873 (*N =* 4 items)			
Scale intraclass correlation coefficient: 0.632, 95%CI (0.575; 0.687), *p < * 0.001^**^			
Residency training program comprises formal education^a^	3.23 ± 1.250 3 (2–4)	3.30 ± 1.203 3 (2–4)	0.776
Curriculum residency training program suits training needs^a^	3.01 ± 1.213 3 (2–4)	3.32 ± 1.198 3 (3–4)	0.063
Opportunity for applied and translational research^a^	2.45 ± 1.277 2 (1–3)	2.72 ± 1.345 3 (1–4)	0.115
Guidance and support for applied and translational research^a^	2.20 ± 1.238 2 (1–3)	2.53 ± 1.394 2 (1–3)	0.088
Overall grade for teaching^a^	2.726 ± 1.04 2.75 (2–3.5)	2.967 ± 1.119 3 (2.25–3.75)	0.089

^a^Numerical variable (from 1-very little so, to 5-strongly agree); mean ± standard deviation; median (IQR), non-parametric Mann-Whitney test. AIC, anesthesiology and intensive care; CI, confidence interval; EM, emergency medicine; IQR, inter-quartile range. Level of statistical significance: ^**^p < 0.01.

There is no significant difference in the overall grade for teaching, and all the four items of the quantitative scale regarding this aspect are balanced (Table 6), with lower opinion of the actually received guidance and support for applied and translational research compared to the formal education (approximately 1-point mean difference on the five-point Likert scale).

On the other hand, the qualitative outcomes in Tables 4, 5 reveal significant differences regarding the actual educational activities (more workshops in AIC and more discussions in EM), evaluation or assessment methods (more OSCE in EM) and their scheduling (stricter and more clearly scheduled in AIC).

### 3.3 Relationship between scales. Overall feed-back

**Figure 2:**
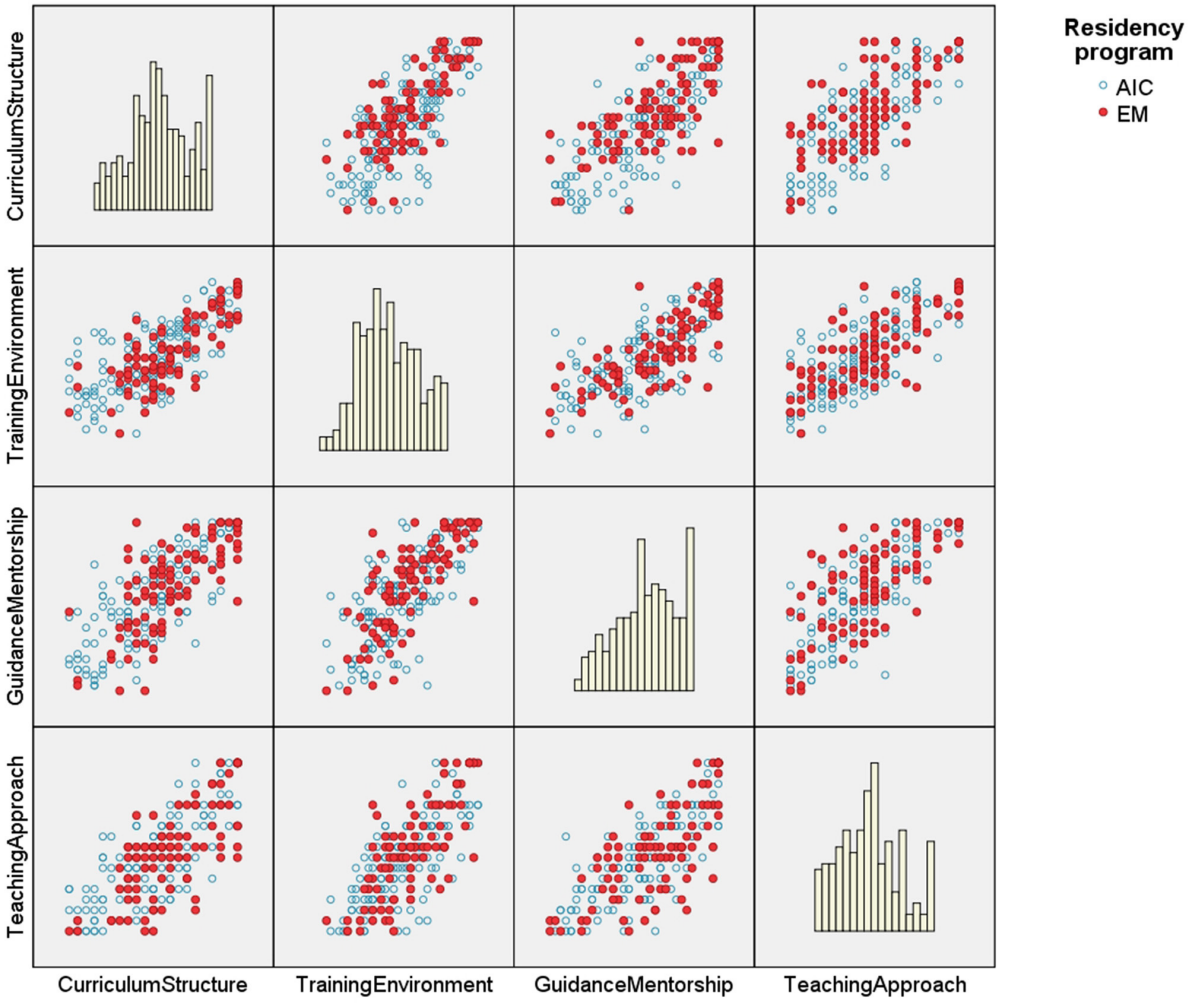
The matrix with two-by-two scatter plots corresponding to the composite measures of the four issues addressed by the main objective. AIC, Anesthesiology and Intensive Care; EM, Emergency Medicine.

The four scales are all two-by-two correlated strongly and significantly. These findings remain consistent for both programs, as can be seen in Tables 7, 8. Figure 2 depicts the matrix with two-by-two scatter plots corresponding to the composite measures of the four issues addressed by the main objective: (a) curriculum and structure of the residency program; (b) training environment, scope, opportunities and complexity; (c) training guidance and mentorship; (d) teaching approach. Histograms depicting the distribution of the values of each composite measurement are on the diagonal of the matrix (Figure 2). Strong and significant correlation can be observed between each two aspects, for both residency programs.

**Table 7:** Non-parametric Spearman correlation between the four scales for AIC residency program.

**AIC (*N =* 137) Spearman correlation coefficients**	**Curriculum and structure of the residency program**	**Training environment adequacy**	**Guidance and mentorship**	**Teaching approach**
Curriculum and structure of the residency program	1	0.715^**^	0.779^**^	0.758^**^
Training environment adequacy		1	0.730^**^	0.731^**^
Guidance and mentorship			1	0.782^**^
Teaching approach				1

^**^Correlation is statistically significant at the 0.01 level (2-tailed). AIC, anesthesiology and intensive care.

**Table 8:** Non-parametric Spearman correlation between the four scales for EM residency program.

**EM (*N =* 98) Spearman correlation coefficients**	**Curriculum and structure of the residency program**	**Training environment adequacy**	**Guidance and mentorship**	**Teaching approach**
Curriculum and structure of the residency program	1	0.763^**^	0.733^**^	0.718^**^
Training environment adequacy		1	0.782^**^	0.737^**^
Guidance and mentorship			1	0.716^**^
Teaching approach				1

^**^Correlation is statistically significant at the 0.01 level (2-tailed). EM, emergency medicine.

**Figure 3:**
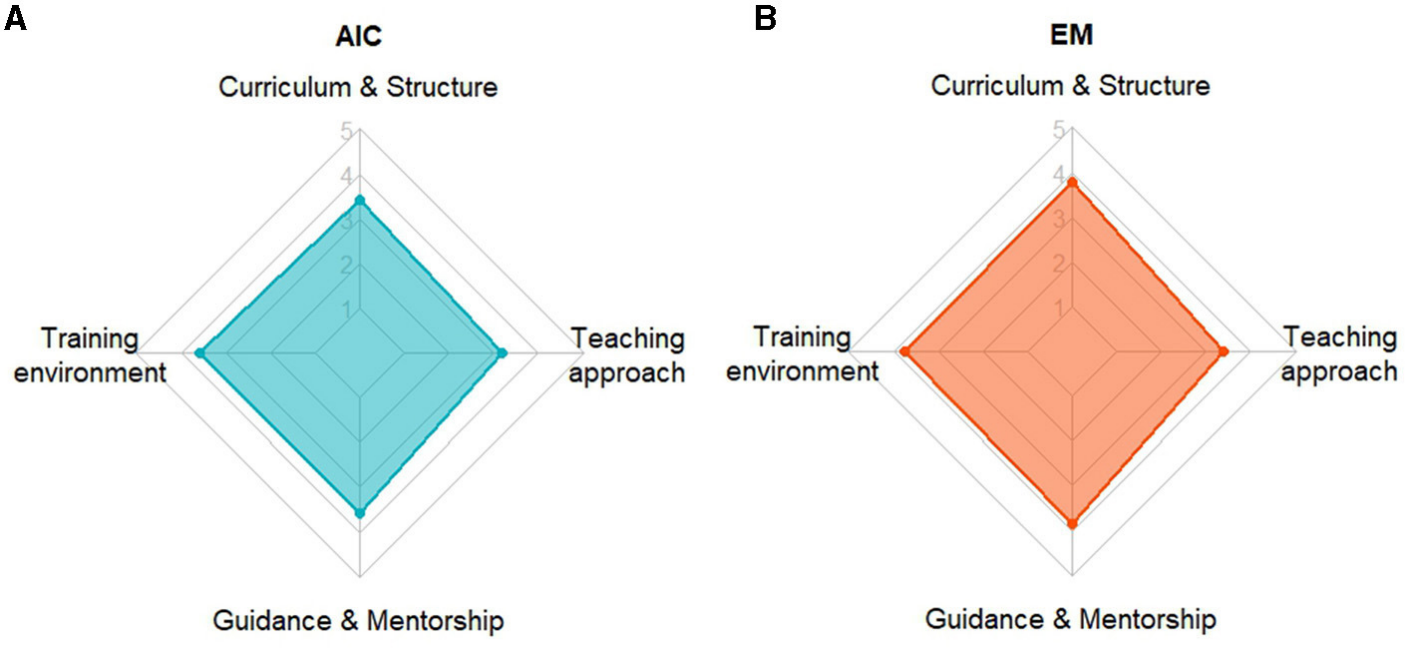
The radar plots with the average values corresponding to the composite measures of the four issues addressed by the main objective: (a) curriculum and structure of the residency program; (b) training environment, scope, opportunities and complexity; (c) training guidance and mentorship; (d) teaching approach. They visualize the comprehensive pattern in residents' feed-back from the two professional groups: **(A)** Anesthesiology and Intensive Care; **(B)** Emergency Medicine. Although neither of the two quadrilaterals is regular, they both are close to rhombi and have equivalent areas.

The radar plots for the scales' average values corresponding to the composite measures of the four issues addressed by the main objective (Figure 3) provide further evidence of both their balanced perception across the four issues and their equivalence in the overall feed-back for AIC and EM residency programs. The areas under the two polygons are similar: 38% and 44% out of the maximum area of the rhombus for AIC and EM, respectively.

### 3.4 Open questions about trainees' opinions

Two independent researchers conducted a thematic analysis on the open-ended questions. An inductive approach was used, aimed to identify any supplementary emergent theme. Differences were resolved by consensus. The results indicate that anesthetists and EM physicians expressed similar opinions of the curriculum design, whether the curriculum was successfully covered or applied, and on what should be changed in terms of training. The results are presented in full in Supplementary file 2.

To a large extent, trainees in both programs considered their respective curriculum as being complex, detailed, and well structured. On the other hand, there were some residents who saw it as incomplete and unstructured, or thought it would benefit from more structure (23 out of 137, and 7 out of 98 respondents for AIC and EM, respectively).

Regarding the curriculum's successful application in practice, some respondents believed this target as achieved to a large extent: 32% AIC, and 16% EM trainees. The remaining respondents considered it applied to a low or very low extent or did not express any opinion. AIC trainees were in favor of having more courses, mentorship, workshops, simulations, case discussions, and more evaluations in the above-mentioned order. EM trainees thought they would benefit more from workshops, case discussions, courses and mentorship (Supplementary file 2).

## 4 Discussion

We conducted an anonymous cross-sectional survey with the primary objective of collecting comprehensive feed-back from AIC and EM trainees. Both fields require practitioners to stay up to date with the latest medical advances and technologies and the same should be true for the design and deployment of the training programs themselves. EM residency trains for rapid and comprehensive response to diverse and time-sensitive clinical scenarios, while AIC training focuses on mastery of complex procedures, precise monitoring, and interdisciplinary collaboration in critical and high-stress situations. The overall feed-back we received from residents of both specialties was equivalent, with differences primarily related to each particular set of professional challenges and educational strategies.

Worldwide, EDs are facing significant professional challenges. According to recent research, EDs experience higher levels of stress for physicians compared to other medical fields. This constant physical and psychological pressure in EM raises concerns about the long-term sustainability of this career for physicians (13–15).

Anesthesiology trainees tend to have higher rates of burnout compared to medical students and practitioners who have completed their training (16). Studies have shown that anesthesiology residents experience professional burnout, while academic anesthesiology faculty members generally report a significantly higher sense of wellbeing than residents (17). In a survey done by The Royal College of Anesthetists, anesthetists of all levels expressed similar sentiments regarding the factors that would contribute to a sustainable and appealing long-term career in anesthesiology (namely, flexibility, feeling valued and maintaining a good work-life balance). They specifically mentioned the benefit of more training opportunities and support for career progression (18).

Our country has little research on graduate medical education and residency training. Additionally, there is insufficient training for supervisors and mentors, and there is no nation-wide formal agreement on effective assessment approaches and instruments. It is important to note that being a skilled and competent professional does not automatically make someone an effective mentor for residents. Acquiring the abilities necessary for medical practice is quite challenging, especially in the fast-paced and highly stressful residency environment of AIC and EM. At the same time, it is vital to prioritize patient safety and ensure that learning experiences occur in a way that does not compromise it, which requires well-trained supervisors and facilitators.

Further understanding of distinctions in residency training programs can help healthcare professionals choose a path aligned with their interests, career goals, and the type of patient care they find most interesting and compelling. Both specialties play critical roles in the continuum of patient care, particularly in the management of acute and critical medical situations.

### 4.1 Curriculum and structure of the residency programs. Scope, opportunities and complexity of the training environment

In our study, almost half of the AIC trainees' opinions regarding the type of curricula are balanced between a European and a national one, while the remaining 42% have no idea about the curricula they are following. On the other hand, just 12% of the EM trainees are not familiar with the type of curricula, half of them thinking they follow a national one and an important number (37.85%) considered their curriculum followed the European recommendations. These observations come into contrast with the answers for the second section of the questionnaire where the overall grade for orientation and curriculum structure was 3 for both specialties.

The requirements and standards for training institutions vary among different European countries. The accreditation conditions for training centers are determined by national regulatory bodies. At the European level, there are established visiting programs and appraisal processes based on the EU Directive on Professional Qualifications and the UEMS (European Union of Medical Specialists) Charta 1997 (19). For anesthesiology, programs such as the “Accreditation of Training in Anaesthesiology and Intensive Care” (ATAIC) are collaboration between European Society of Anaesthesiology and Intensive Care (ESAIC) and European Board of Anesthesiology (EBA) (19).

The European Training Requirement (ETR) for EM underwent a thorough review and update to align with the current practices and the evolution of the specialty across Europe. Approved by the UEMS Council in April 2019, this revision builds upon the European core curriculum initially published in 2002. ETR update process initiated in January 2022 involved a comprehensive examination of the clinical practices and specialized skills within emergency medicine (20). Representatives from the UEMS section and Board for Emergency Medicine, along with EUSEM education committee and the Young Emergency Medicine Doctors' group actively participated in this collaborative effort.

The way trainees perceive their educational environment is an important factor in determining how successful the curriculum implementation is (21). The Romanian trainees also provided highly correlated feed-back on all the four rated aspects: curriculum and structure of the residency program; the training environment scope, opportunities, and complexity; training guidance and mentorship; teaching and assessment approaches. Respondents demonstrated a comparable level of agreement regarding the overall opinion on educational environment for the two specialties. Training sites are considered adequate in terms of educational content and complexity of cases encountered in the department by all residents. Overall, the relationship and the support the trainees receive on the other wards during the rotations are perceived as appropriate. However, the residents from both specialties gave lower ratings to the relationships with the faculty across departments, particularly concerning cross-departmental curriculum compliance. In contrast, there were divergent opinions on the suitable pace for progressive responsibility development between the two specialties: EM residents feel they are entrusted with more significant responsibilities compared to AIC residents (3.92 ± 1.071 vs. 3.54 ± 1.182; *p* = 0.014). Additionally, EM trainees believe they receive greater support from the rest of the staff in their respective department (3.43 ± 1.316 vs. 2.78 ± 1.229; *p* < 0.001).

### 4.2 Training guidance and mentorship

The literature strongly emphasizes the numerous advantages of mentorship, such as enhanced academic and personal productivity, career progression, higher retention rates among trainees, improved educational abilities, boosted self-assurance, increased grant funding, and success in academic pursuits (22–26).

According to EBA UEMS, requirements for AIC trainers include: having competence level D in the designated training area, allocating adequate time for training assignments, possessing knowledge about the UEMS ETR, maintaining a positive attitude toward clinical training, demonstrating expertise in didactic teaching, and exhibiting a clear commitment to providing theoretical instruction and practical guidance to trainees across the entire spectrum of clinical practice (19).

In Romania, a training supervisor for AIC is required to be a medical specialist with a minimum of 5 years of experience in the AIC specialty. They are responsible for overseeing a maximum of five residents (irrespective of trainees' year of residency). The supervisor monitors the residents' progress, reviews and approves the completion of the practical skills in the training curriculum, and coordinates seminars and discussions. These education sessions, lasting a minimum of 4 hours per week, cover specific topics outlined in the training curriculum, including case presentations, articles, and updates relevant to the field (27).

According to the national curriculum for EM training, the residents' progress should be monitored and they should be guided through the training modules, to ensure they learn the necessary knowledge and practical skills. Professionals in charge of training must meet certain eligibility criteria: be a consultant or specialist in EM with residency in EM or AIC, and at least 5 years of experience in ED. In addition, they must perform on-call duties within the ED where the residents are registered (8).

When asked if they had a supervisor, 80% of EM trainees gave a positive answer while 20% did not know or did not have one. More than half of the trainees had a good opinion of their mentor, considering (s)he had specific training for teaching. However, less than half of them (40%) declared having monthly meetings with their mentors for teaching sessions and progress evaluation. Just 64% of AIC trainees knew they had a mentor, and < 40% believed their mentors had previous training (any of “train the trainer” courses or didactics). In this specialty, the residents considerably missed teaching and supervision, with only 13.9% of them having monthly meetings with the mentors for assessing their progress, and < 30% of them declared having monthly meetings for teaching activities.

In a study by Craig et al. (10), most EM trainees believed they needed more direct supervision at the bedside and they valued the feed-back from their mentors and program's director. Additionally, they felt the feed-back was too much focused on negative aspects of their performance and was rarely based on their observed behavior (10).

Ergun et al. (28) studied perceptions regarding mentorship among Canadian anesthesia residents. Seventy-nine percent of surveyed residents expressed their agreement on the significance of mentorship for achieving overall success in the field of anesthesiology. Obstacles in establishing successful mentorship included ineffective connection between mentors and mentees (74% of respondents), mentors' time constraints (70% of respondents), and scarcity of mentors who actually shared similar personal and professional aspirations with the mentees (61% of respondents). Also, residents highlighted the absence of structured meetings (62% of respondents) and missing clearly defined objectives in their mentorship programs (58% of respondents) as additional barriers (28).

Trainee-centered approaches and mentor-mentee compatibility are crucial for successful mentoring, as is timely and critical evaluation of trainees' progress (29–32).

### 4.3 Teaching and assessment approaches

Perception of overall teaching approach had no significant differences between AIC and EM in our survey. However, when we analyzed the qualitative data, AIC trainees had more simulations and more workshops than EM residents, while case discussions and case presentations were more frequently mentioned in the EM training. Still, the frequency of formal education was low in both AIC and EM.

Research of Nunes et al. (33) emphasized the importance of in-service training during medical residency. They suggested that such training should effectively combine teaching and learning, setting a high standard for professional development. The goal is to provide medical residents with opportunities to expand their knowledge, enhance their attitudes and skills, and cultivate specific proficiencies. Students and trainees are more committed and have better performance when they can see the explicit connection between the educational activities or requirements and their future professional expertise (30, 32, 34). Lower rating for the quality of theoretical education in anesthesiology residency was reported when compared to other educational activities (30–32, 35–37). Several possible explanations have been considered, such as absence of comprehensive training programs and even mentors' qualifications (35).

The trainees in our study also suggested that more simulations, workshops and case discussions might improve their training. Although this might be a bias from the online education during the COVID-19 pandemic, we would rather see it as a consequence of these activities missing in the actual residency programs. We acknowledge that online learning can never fully replace hands-on teaching (30–32, 38–40), but the COVID-19 pandemic also brought challenges related to patient bedside learning (41).

Simulation activities go beyond just improving technical skills in a safe setting. They provide actual experiences that are highly effective in retaining knowledge and have the potential to generate behavioral changes, such as in leadership, communication, and resource management. This, in turn, enhances patient safety (37, 42), and can bridge theoretical lessons with real-world clinical practice, particularly in helping junior doctors handle emergency situations (43). Simulation programs positively impact non-technical and behavioral issues, influencing the learning process in both EM and anesthesiology (31, 44–46). Moreover, highly immersive and interactive educational solutions are emerging, such as 360-degree video training, telementoring and metaverse approaches (36, 38, 47–50).

In our analysis of scales' relationship, we noted a consistent pattern of high statistical significance in pairwise correlations across all respondents. This pattern held true for both AIC and EM curricula. From this robust pair-wise correlation we can infer that effective resident orientation and high-quality mentorship contribute to a positive perception among residents regarding the learning environment and methods.

In a study conducted by Gonzales et al. (51) on mentorship of residents in anesthesiology, residency program directors identified career planning, professionalism, and maintaining a balance between personal, career, and family commitments as crucial areas for effective training. These principles hold true for residents in EM as well, considering that with the support of residency community, young doctors can nurture resilience and mitigate the risk of burnout (52). By cultivating a culture of wellness, AIC and EM residency programs can create an environment that supports residents' physical and mental wellbeing, fosters learning, and ultimately enhances their efficiency and success throughout prolonged training periods. Creating a supportive and collegial atmosphere where residents feel comfortable seeking help and support from their peers and supervisors improves their learning experience (53–55).

The visual representations of the responses of residents from AIC and EM highlighted the similarity in opinions. The only discrepancies were minimal and rather qualitative, stemming from the specific details of the two residency programs. Based on the results, we strongly recommend improving the quality of the trainer-trainee relationship, irrespective of specialty, and employing interactive technology-based educational solutions, such as augmented reality, virtual reality, or metaverse. They would help further tailoring the training experience, particularly in the context of needed feed-back, guidance, and mentorship.

### 4.4 Strengths and limitations of the survey

One important strength of the study consists in collecting and processing feed-back from all the 11 Romanian centers that have residency programs in AIC and EM, thus setting a reliable basis for further longitudinal studies. Although the geographical distribution of respondents was non-uniform, and non-response bias might affect the results, the moderate response rate and having concrete effect size for the scale measurements is an important step forward toward power calculation in future studies' design.

We also acknowledge the limitation rooted in the very design of the study, which collected anonymous self-reported data, with no objective validity confirmation and no control of respondents' background. Moreover, respondents were in different years of residency and had mixed experience (some respondents might already have a medical specialty). Future studies should be stratified according to trainees' level of experience.

Common method bias (CMB) and its associated effect of common method variance (CMV) is another major concern to be acknowledged, due to the cross-sectional design and one-time single administration of the questionnaire to each professional group of residents. The survey questionnaire was designed to limit the shared variance and to control the method biases by alternating the subscales with factual questions and categorical options to be checked on the one hand and distributing the scale questions referring to the same axis/issue across the questionnaire, on the other hand.

## 5 Conclusions

AIC and EM residency training in Romania have national curricula aligned with European standards, but future improvement should seek better deployment in practice, particularly with regard to professional guidance, mentoring, and constant feed-back to the trainees. Benefits from this analysis also include supporting shared decision-making for integrating metaverse solutions into medical training to provide opportunities for highly immersive and interactive educational experiences, particularly valuable for critical care trainees.

In addition, with the worthwhile feed-back we received, this investigation can serve as a pilot study for further multicenter longitudinal research, such as comparative analyses of training programs across different healthcare systems.

## Data availability statement

Data set is available in full on FigShare (https://doi.org/10.6084/m9.figshare.25997302.v1) and further inquiries can be directed to the corresponding author.

## Ethics statement

The studies involving humans were approved by the Decision of the Local Scientific Research Ethics Commission of the County Emergency Clinical Hospital Pius Brinzeu Timisoara no. 432/16.01.2024. The studies were conducted in accordance with the local legislation and institutional requirements. The participants provided their written informed consent to participate in this study.

## Author contributions

CB: Conceptualization, Data curation, Investigation, Methodology, Project administration, Validation, Visualization, Writing–original draft, Writing–review & editing. AP: Conceptualization, Data curation, Methodology, Project administration, Supervision, Validation, Visualization, Writing–original draft, Writing–review & editing. DL: Conceptualization, Data curation, Formal analysis, Project administration, Software, Supervision, Validation, Visualization, Writing–original draft, Writing–review & editing. AM: Conceptualization, Data curation, Investigation, Project administration, Software, Writing–original draft, Writing–review & editing. OB: Methodology, Software, Supervision, Visualization, Writing–review & editing. MP: Investigation, Methodology, Resources, Visualization, Writing–review & editing. CT: Investigation, Resources, Software, Visualization, Writing–review & editing. MB: Data curation, Investigation, Resources, Visualization, Writing–review & editing. OM: Supervision, Validation, Visualization, Writing–review & editing. DS: Supervision, Validation, Visualization, Writing–review & editing.
