# Supplementary material for: Residency training programs in anesthesiology, intensive care and emergency medicine: from curriculum to practice
Source: Front Med (Lausanne). 2024 Jun 26;11:1386681. doi: 10.3389/fmed.2024.1386681 (PMC11264376; doi:10.3389/fmed.2024.1386681)
Supplement: Supplementary file 1 [file Table_1.pdf]

Residency training programs in anesthesiology, intensive care and emergency medicine: from curriculum to practice (doi: 10.3389/fmed.2024.1386681)

Supplementary file 1: The survey questionnaire

This survey is addressed to all Emergency Medicine (EM)/Anesthesia and Intensive Care (AIC) trainees undergoing a residency program. The aim of this survey is to identify areas for faculty development and ways to better convey into practice the EM/AIC curriculum.

Please take this survey if you currently are an EM/AIC trainee.

We would greatly appreciate if you would share this survey among your peers.

I consent to the study:

- o Yes
- o No

REGION/UNIVERSITY/ TOWN OF THE RESIDENCY PROGRAMME

GENDER

A. Induction to the residency programme

to a low extent 1 2 3 4 5 to a large extent

A1. You are provided with appropriate guidance for your entire training program.

A2. You are given proper goals, objectives to be achieved during your residency years.

A3. You are correctly oriented and presented on departments, facilities, teaching faculty, teaching equipment.

B. Curriculum

B1. Your specialty training program is based on:

- o The curriculum endorsed by EUSEM/ESAIC
- o A national speciality curriculum
- o We do not follow a specific speciality curriculum
- o I don’t know

B2. The curriculum has well defined aims and objectives

to a low extent 1 2 3 4 5 to a large extent

B3. The program is well structured and organized

to a low extent 1 2 3 4 5 to a large extent

C. Work environment

C1. How many different EM/AIC departments do you rotate during your training programme?

- 1 - 2
- 3 – 4
- 5 or more

C2. Your training sites are adequate in terms of number and educational content.

to a low extent 1 2 3 4 5 to a large extent

C3. The EM/AIC patient population case mix is adequate in number and variety.

to a low extent 1 2 3 4 5 to a large extent

C4. You have ample opportunity to develop procedural, teaching and leadership skills.

to a low extent 1 2 3 4 5 to a large extent

C5. You are provided with progressive responsibilities appropriate to the level of training.

to a low extent 1 2 3 4 5 to a large extent

D. Mentorship

D1. Throughout your specialty training in EM/AIC, is there a designated consultant/specialist who acts as your supervisor for the entire training period?

- Yes
- No
- Don't Know
- Not Applicable

D2. If the previous response was yes, do you possess information about whether your supervisor has undergone specific training in medical education before taking on their role?

- Yes
- No
- I would not know
- Not applicable

D3. The number and availability of your supervisors are sufficient to meet your training objectives.

to a low extent 1 2 3 4 5 to a large extent

D4. How frequently do you have meetings with your assigned supervisor to assess and discuss your progress?

- o At the beginning, mid-way through and at the end of each rotation (once every 3 months)
- o Once per year of training
- o Less than once per year
- o Never
- o Not Applicable

D5. How frequently do you engage in educational meetings with your assigned supervisor?

- o Monthly
- o 1-2 times per year
- o Never
- o Not applicable
- o Others

D6. Your clinical work is under constant supervision, ensuring proper guidance and teaching by your supervisor or other senior doctors within the department.

to a low extent 1 2 3 4 5 to a large extent

D7. Your supervisors are actively performing ”bedside teaching” for trainees.

to a low extent 1 2 3 4 5 to a large extent

E. Teaching and educational activities

E1. Does your training program incorporate any structured educational sessions or formal teaching elements?

to a low extent 1 2 3 4 5 to a large extent

E2 . What types of educational activities are offered by your training institution? Please select all that apply.

- o Face-to-face lectures (virtual or in a classroom)
- o Practical skills workshops
- o Case based discussions (with trainee as team lead)
- o Case presentations performed by trainees
- o Simulations (low to high fidelity)
- o Journal clubs
- o Leadership training
- o Other (please specify)

E3. Which is the periodicity of the educational activities?

- Every week
- Every month
- Every 4 months
- Twice a year
- Once a year
- Never

E4. In your opinion, to what degree does the teaching program provided by your institution fulfill your educational needs?

- To a very small extent
- to a small extent
- to a large extent
- to a very large extent
- neither small, nor large extent

E5. From your perspective, which teaching activities do you find most valuable and, consequently, think should be arranged more frequently?

- Face-to-face lectures
- Practical skills workshops
- Case based discussions (with trainee as team lead)
- Case presentations performed by trainees
- Simulations (low to high fidelity)
- Journal clubs
- Leadership training

E6. Is attendance to national/international resuscitation courses compulsory (i.e. ALS, ACLS, APLS, ATLS, ETC)?

- Yes
- No

F. Evaluation

F1. Which methods of examination are employed? Please select all that are applicable.

- o Multiple Choice questions
- o Short Answer Questions
- o Objective Structured Clinical Examination
- o Critical appraisal of scientific literature
- o Interview
- o Other (please specify)

F2. How are the exams scheduled throughout your training program?

- o At the end of every year of training
- o At the end of every rotation
- o Ocasionally
- o Never
- o Other (please specify)

G. Research

G1. I have been offered numerous opportunities to foster my knowledge and passion for research in the field of EM/AIC.

to a low extent 1 2 3 4 5 to a large extent

G2. I have received guidance and support in actively participating in research activities.

to a low extent 1 2 3 4 5 to a large extent

H. Departmental and interdepartmental support/relations

H1. Relationship with other departments is optimal.

to a low extent 1 2 3 4 5 to a large extent

H2. Collaboration with other departments is established to ensure the achievement of the required teaching standards throughout rotations.

to a low extent 1 2 3 4 5 to a large extent

H3. Other departments teach the required curriculum during rotations.

to a low extent 1 2 3 4 5 to a large extent

H4. Support of ED/ICU department staff is optimum during rotations.

to a low extent 1 2 3 4 5 to a large extent

I. Resources, infrastructure

I1. The infrastructure of the emergency department (ED) / intensive care unit (ICU) provides ample space and facilities to fulfill educational needs.

to a low extent 1 2 3 4 5 to a large extent

I2. The infrastructure of the hospital provides ample space and facilities to meet the educational needs effectively.

to a low extent 1 2 3 4 5 to a large extent

J. Program director(PD)

J1. The PD gives feedback about your progress and deficiencies.

to a low extent 1 2 3 4 5 to a large extent

J2.The PD addresses all the issues and problems which are relayed to him.

to a low extent 1 2 3 4 5 to a large extent

J3.The PD plays effective role in dealing with educational and teaching issues.

to a low extent 1 2 3 4 5 to a large extent

J4. The PD plays a crucial and efficient role in handling matters pertaining to services, schedules, education, faculty, and rotations in other departments.

to a low extent 1 2 3 4 5 to a large extent

J5. The PD is easily accessible and promptly responds whenever you reach out for assistance.

to a low extent 1 2 3 4 5 to a large extent

K. Your opinion

Describe in 3 words/group of words your opinion regarding the EM/AIC curriculum

Describe in 3 words/group of words your opinion regarding the convey in practice of the EM/AIC curriculum

Describe in 3 words/group of words your opinion regarding what should be changed in EM/AIC training.

The feed-back collected brought insights into five areas of the junior doctors' training: curriculum and structure of the residency program (A, B); the training environment scope, opportunities and complexity (C, H, I); training guidance and mentorship (D, J); teaching approach (E, F, G); overall trainees' perception (K).
