# Supplementary material for: Residency training programs in anesthesiology, intensive care and emergency medicine: from curriculum to practice
Source: Front Med (Lausanne). 2024 Jun 26;11:1386681. doi: 10.3389/fmed.2024.1386681 (PMC11264376; doi:10.3389/fmed.2024.1386681)
Supplement: Supplementary file 2 [file Table_2.pdf]

Residency training programs in anesthesiology, intensive care and emergency medicine: from curriculum to practice (doi: 10.3389/fmed.2024.1386681)

Supplementary file 2: Thematic analysis of open questions about trainees' options

**Variable**	**AIC** (N=137)	**EM** (N=98)
Opinions regarding the curriculum		
a lot of useless rotations	–	2
adequate	17	–
complete	6	–
complex, extended, detailed	33	14
could be more structured	6	7
incomplete, unstructured	17	–
is not applied in practice	11	5
just perfect	–	6
needs more mentors' involvement	3	7
needs more practical skills teaching	–	3
needs rotations change (some should be added, others excluded)	2	8
no opinion	15	32
not known	4	–
outdated	6	2
should include weekly courses	4	–
very well structured	5	–
well structured	8	12

Opinions regarding the conveyance into practice (whether the curriculum is successfully covered or applied)		
could benefit of extensive change	–	10
dependent on the mentors	–	5
no opinion	19	35
to a large extent	36	13
to a low extent	25	8
to a very large extent	8	3
to a very low extent	49	24
Opinions on what should be changed		
more courses	36	7
more simulations	16	5
more workshops	22	18
more case discussions	14	8
more case presentations	13	–
more evaluations	10	1
more mentorship	26	6
more guidelines	2	1
everything	7	5
nothing	3	5
no opinion	18	31
Other (better organized)	22	11
